# Worksite Health Promotion in Six Varied US Sites: Beta Testing as a Needed Translational Step

**DOI:** 10.1155/2011/797646

**Published:** 2011-04-07

**Authors:** Diane L. Elliot, Kerry S. Kuehl, Linn Goldberg, Carol A. DeFrancesco, Esther L. Moe

**Affiliations:** Division of Health Promotion & Sports Medicine, Oregon Health & Science University, CR110 3181 SW Sam Jackson Park Road, Portland, OR 97239-3098, USA

## Abstract

*Background*. Dissemination of health promotion interventions generally has followed an efficacy, effectiveness to full scale paradigm, and most programs have failed to traverse that sequence. *Objective*. Report national dissemination of a health promotion program and juxtapose sequential case study observations with the current technology transfer literature. *Design*. Multiple department-level case studies using contact logs, transcribed interactions, augmented with field notes and validated by respondent review; at least two investigators independently generated site summaries, which were compared to formulate a final report. *Results*. Adoption was facilitated with national partners and designing branded materials. Critical site influences included departmental features, local champions, and liaison relationships. Achieving distal reach and fidelity required sequential process and program revisions based on new findings at each site. *Conclusions*. Beta testing to redesign program elements and modify process steps appears to be a needed and often ignored translational step between efficacy and more widespread dissemination.

## 1. Introduction

Few wellness domains are as timely as is achieving a healthy lifestyle. Currently, less than five percent of US adults simultaneously attain the national dietary, physical activity, and body weight objectives [1]. Worksites have been identified as key channels for health promotion. They are natural settings for environmental restructuring and altering social norms, leading to outcomes that benefit both workers and employers [2]. However, as in many fields, a wide gap exists between investigators and occupational practitioners. Evidence-based interventions often are not used, and those that are used frequently have not been studied [3].

The PHLAME (Promoting Healthy Lifestyles: Alternative Models' Effects) study was an NIH-funded prospective randomized assessment of two worksite health promotion paradigms among firefighters. Despite public perceptions, firefighters are a high-risk group, with an increased prevalence of obesity, hypertension, dyslipidemia, certain malignancies, and chronic musculoskeletal complaints [4]. Their elevated cardiac risk profile combined with periodic intense work effort may account for heart attacks being the leading cause of firefighters' on-the-job deaths [5], compared to approximately 10 percent for other first responders [6].

The original PHLAME study involved two models for worksite wellness: (1) a team-centered, peer-led scripted curriculum and (2) individual counseling using motivational interviewing (MI) techniques, versus (3) a testing and results only control condition [7]. At one-year followup, both interventions significantly increased healthy dietary behavior (*P* < .005), fitness parameters (*P* < .05) and general well-being (*P* < .01), and resulted in less weight gain (*P* < .05). Both also resulted in an immediate reduction in work-related injuries [8]. 

**Figure 1:**
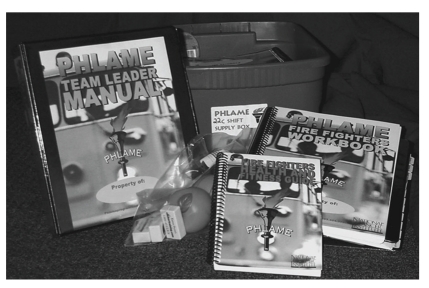
PHLAME team curriculum components.

The team-centered intervention was less expensive than counseling, and that format also is a natural fit for firefighters' work structure, as a shift at a fire station is an existing team. The PHLAME team-centered curriculum is 12, 45-minute peer-led sessions. One shift member uses a Team Leader Manual with explicitly scripted lesson plans, and teammates use corresponding Workbooks. Participants each receive a pocket-sized Guide with expert, firefighter specific content. Each team session is composed of three to six interactive activities, with core components involving nutrition, physical activity, and energy balance. Between session materials are stored in a Team Box, which also stores an accompanying Family Manual and program props (Figure 1).

Because of its positive outcomes, NIH funding was continued to implement the team-centered program in other fire departments across the US and to follow the original cohort for durability of change [9]. We sought to study the dissemination process across departments using a multiple case study format and compare those observations with the technology transfer literature.

## 2. Methods

### 2.1. Recruiting PHLAME Departments

The International Association of Fire Fighters (IAFF) is one of the largest AFL-CIO unions; it was an important partner in recruiting PHLAME dissemination sites. The organization arranged for a PHLAME presentation at an annual IAFF Symposium, where we distributed recruitment materials. 

The marketing literature documents the importance of a product branding [10], and in anticipation of recruitment, we developed a consistent product brand, logo, and promotional materials highlighting aspects identified as augmenting marketing success [11]. We included PHLAME's positive outcomes but recognized efficacy alone is rarely sufficient to generate participation [12]. Accordingly we produced a three-minute promotional DVD, using testimonials from the original study participants to further promote interest [13], linked to an informational website (http://www.phlameprogram.com/).

### 2.2. Initial Contacts and Case Selection

We received more than 30 inquires from fire departments across the US. Communicating with departments was a time-consuming process; on average, four contacts per site were needed to exchange information and answer questions. Departments were selected based on sustained interest and a verbal commitment to participate. From 15 interested sites, 8 (maximum feasible with our funding) were selected to provide a range in sizes and locations.

As with most worksites, fire departments have a hierarchy of decision makers, who differ from the employees ultimately participating in worksite wellness. Once identified as a dissemination department, a second level of communication was required. We visited each department from two to six times, to personally describe the program and meet with the Chief, Wellness Committee, union officials, and other decision makers. Two sites elected not to participate while finalizing plans and were replaced. In those locations, contract negotiations and turnover in key personnel could trump initial administrative and department-wide enthusiasm. “Turbulence” has been used to describe occurrences that adversely affect implementation of drug use prevention curricula [14], and a similar term would apply to worksites' adoption of health promotion programs.

### 2.3. Case Study Methodology

Case studies are a common strategy in education, political science, and business settings [15]. They can provide information concerning explanatory links, rather than event frequencies, and are a means for empiric inquiry about contemporary phenomenon, especially when the boundaries between the phenomenon and context are blurred. Multiple sequential case observations were selected for pragmatic and theoretical reason. Time to installation differed among departments, and multiple case studies allowed contrasting results for predictable reasons, comparable to a series of experiments [15]. Our hypothesis was that our health promotion program would be altered over time in a direction that would allow for a more robust subsequent effectiveness testing. In reporting the case study findings, within space limitations, we have adhered to recommendations of the STROBE Statement [16].

### 2.4. Qualitative Assessment of Dissemination Sites

Once the program was adopted and installed, the departments were assessed during follow-up visits using interviews of key informants, such as the wellness coordinator and union head, and focus groups of shift members at fire stations. Purposeful sampling was used to involve at least some members from all work groups initiating the PHLAME team program, along with selected firefighters from work units not participating [17]. The interactions used a semistructured interview guide organized around contextual variables and the concepts typically identified as relating to diffusion of technology (Table 1).

Conversations were recorded, transcribed, and augmented with field notes concerning circumstances and additional observations. The qualitative information was read and coded to identify themes and sorted based on surfacing key issues and shared concepts. Findings were validated by contacting selected respondents and asking them to review a transcript summary and/or notes for accuracy. Compiled qualitative findings for each site were reviewed by at least two investigators, who generated independent case reports, which were then compared for content and implications, and a final case summary prepared for each site [15]. The Institutional Review Board of the Oregon Health & Science University approved the study procedures.

## 3. Results

### 3.1. Dissemination Case Studies

**Case 1**

Eugene, Oregon was selected as the initial site because of its close proximity and its first-hand awareness of the PHLAME study. The department has approximately 200 employees and 11 stations. Eighteen of 30 potential PHLAME teams had stable membership and were enrolled. Although the department administration and wellness coordinator knew about the program, we overestimated penetration of information to line firefighters. Firefighter participants reported that not knowing more about the program before beginning the team sessions generated suspicion and reactance among coworkers. Reactance or an opposition to actions perceived to threaten behavioral freedoms can cause individuals to adopt a contrary attitude. Most firefighters are male, and because reactance is greater among men than women [23], our population especially may have been vulnerable to inadequately informing them about the program.

PHLAME's 12 sessions originally were formatted to be distributed over 12 months, and clusters of weekly sessions were bridged by competitions among team members to maintain a program presence during breaks. However, Eugene teams found it difficult to sustain members' enthusiasm and momentum during the weeks without meetings, and teams often did not resume sessions. Only 3 of 18 Eugene teams completed the majority of sessions, and that finding was in marked contrast to three-quarters of teams completing most sessions in the initial closely monitored PHLAME study [7]. This finding and those in Denver (Case 2) led to a major revision of the program to consolidate the sessions into 12 approximately weekly meetings.

**Case 2**

The Denver South Metro Fire Department (DSMFD) was selected as a mid-western site, comparable in size to the fire department in Eugene, Oregon. It had no health promotion activities or wellness committee, but its wellness officer was a strong local program advocate. We worked with him to distribute program information to all department members, following which he held a general vote of all firefighters about participating. Although the majority voted to participate, rather than mandatory involvement, we advocated maintaining the program as voluntary, both to meet our Human Subjects requirements, and because it is supported as more successful by the human resource literature [24].

Using this strategy, 22 of 30 potential teams began the program, and the majority completed several months of the original 12-month format. Those teams not enrolling generally reported that they felt they already knew the material or the senior officer and/or assigned team leader were not supportive of the program. As in Case 1, continuing momentum across breaks was a challenge. In response, during a six-month site visit, we initiated a rekindling curriculum. Rekindling involved a start-up session and instructions to complete the remaining sessions at weekly intervals, without breaks. With that revised schedule, most teams reinitiating the program completed the majority of sessions. That success affirmed the need to revise the program into weekly session format.

**Case 3**

The Sacramento City Fire Department (SCFD) is a large organization with 22 stations and 571 firefighters, comparable to Portland, Oregon, which was an original PHLAME study site. Although it had an exercise physiologist on staff, departmental officials expressed a particular interest in the nutrition aspects of PHLAME. The physiologist was a vocal local advocate but not an administrative leader, and he was not supported by an established wellness committee. His role would be better described as an organizational rascal than an opinion leader. The dissemination literature describes rascals as individuals who work behind the scenes and maintain enthusiasm among personnel ultimately implementing the program [25, 26].

Working with a larger organization necessitated a prolonged adoption process and five site visits. Adoption and implementation often have been characterized as a two-stage process [27]. We found it a more protracted sequence of many layers of “choosers,” few of whom would become the end users. Once participation was approved, we selected eight teams by identifying work groups demonstrating sustained interest during the adoption process, including two teams of administrative personnel. Because the latter were highly visible, the “early adopters” administrator assisted in enrolling a second wave of 15 additional teams, most of which completed the revised weekly PHLAME session format. Visible support of administrative personnel has been identified as an important factor in school-based program adoption [28], and we continued to target those groups in later departments.

**Case 4**

Montgomery County, Maryland Fire and Rescue (MCMFR) is a large (39 stations), well-funded department with an established wellness program. Its two wellness officers wanted to add PHLAME to their ongoing health promotion activities. While we recognized the challenges in working with a large department, the potential of the MCMFR Chief as a program champion within national firefighter organizations, lead us to enroll MCMFR. To provide program information, we worked with local staff to develop a site-specific informational DVD, which was delivered to all stations and attached to the department's website. We identified interested work groups and began the program with 15 participating shifts. However, following installation, many MCMFR firefighters were deployed to a national disaster area, which disrupted teams' membership. The only team completing all sessions were fire administration personnel, whose teams remained intact.

An important aspect of the MCMFR experience was the department's strategic location near the IAFF headquarters in Washington, D.C. As a result, IAFF officials were able to directly observe and experience the PHLAME program. Importantly, they reported that seeing it in action facilitated their continued support, and initial IAFF assistance advanced to program endorsement and inclusion of the PHLAME team program in the IAFF Wellness Directive (http://www.iaff.org/hs/well/index.htm).

Approximately one year after the initial installation, a second wave of MCMFR teams were identified. Monitoring a program is known to improve implementation [29], and during our original PHLAME intervention study, research staff closely followed teams' progress. To intensify efforts to achieve success, we hired two local program liaisons to visit stations, assess adherence, and obtain follow-up information. Despite liaisons' monthly visits, no team completed more than half the sessions. Focus group feedback indicated that the MCMFR liaisons may have created more reactance than augmenting program adherence. For MCMFR, the liaisons were two fourth-year George Washington University medical students interested in worksite wellness. Neither had experience as firefighters or direct ties to MCMFR, and it appeared that they never established the collaborative relationship needed to facilitate program use [19].

**Case 5**

The San Diego Fire Department (SDFD) received a Federal Emergency Management Administration (FEMA) grant to enhance wellness activities and independently contacted us about implementing the PHLAME team program. Their FEMA grant supported one full-time and two part-time positions for wellness activities. SDFD is a large department with 1133 firefighters. Because of FEMA funding, the department needed to document changes in behavior, and we provided simple anthropometric measures, fitness self-assessments and a validated fruit and vegetable intake screener with online scoring for use as a dietary pre- and postmeasure [30].

SDFD was the first site where, from the outset, we used a paid local liaison to monitor teams' progress. In this setting, the liaison was a San Diego resident, who was not an SDFD employee but had friends in the department and experience with the PHLAME format. She was charged with periodically visiting stations to learn about their experience and provide assistance when needed. Eight of 17 PHLAME teams completed the majority of the sessions, and noncompletion primarily related to scheduling conflicts, such as the team leader being injured and wildfires necessitating deploying firefighters off site. The overall program success was further evidenced by SDFD requesting additional materials and with minimal assistance, initiated the program in a neighboring smaller fire department. The PHLAME program positive outcomes assisted SDFD wellness program's continuation after the FEMA grant ended.

**Case 6**

The sixth dissemination site was the Indianapolis Fire Department (IFD). IFD is a large department with 34 stations. An individual involved in the city's employee wellness activities spear-headed adopting PHLAME, and we hired that person as the PHLAME liaison. We worked with him and the department's wellness committee to identify a three-month interval with a minimum of conflicts with vacations, training activities, and offsite deployments. Thus, we were able to combine a program champion as our paid liaison and strategic scheduling to maximize PHLAME team member consistency. Using those techniques, three-quarters of teams completed the majority of sessions, which was comparable to the success of the original PHLAME team program.

## 4. Discussion

Rogers differentiated technology transfer from diffusion of ideas, with the former needing a directed process, rather that the entropy that guides diffusion [31]. While the PHLAME program possessed many features identified as aiding translation, obtaining the users' perspectives and defining contextual influences appeared instrumental for facilitating its reach. Bridging the gap between efficacy and distal use required revisions that were both process-oriented and programmatic. 

First, sequential beta testing appeared to be a critical step. Beta testing refers to nonrandomly selected adopters “trying out” a product, reporting on their experience, and using those findings to revise the product, and although the business literature recognizes it as a necessary step in product development [32], it often is omitted from models of translation [33]. While beta testing usually refers to individual testers, it also could apply to organizations' product use [34]. Others have noted that lack of a market perspective may have limited successful translation [22, 35]. More attention to these factors may facilitate application of the established translation metrics, such as reach, adoption, and implementation [36]. 

Recruitment and enrollment are instrumental components for efficacy trials and equally so with dissemination studies. Strategies that appeared to facilitate site enrollment included accessing an existing national organization's communication channels [37] and designing branded tailored recruitment materials. Communication with sites, including repeated personal visits, was more extensive than anticipated, and we confirmed the importance of face to face contact to appropriately fit innovations into target organizations [38]. 

As evidenced by the experience in Denver and Indianapolis, midlevel individuals were the most effective change or linkage agents and appeared best able to traverse the boundary between the administration and end users. In general, the PHLAME content remained durable throughout its sequential use. The single feature changed was session scheduling, going from a year long to three-month format. The rationale for the initial duration was the potential of more durable positive outcomes. However, review of the Cancer Control P.L.A.N.E.T. website of evidence-based diet and physical activity programs reveals a range of intervention lengths, many having three months and less duration (http://cancercontrolplanet.cancer.gov/). Although redesign for feasibility may be bargaining away from effectiveness, the original PHLAME mediation results provided guidance about what program aspects were and were not negotiable [39]. 

Our qualitative case study methods have limitations, with the potential for response bias among both participants and those coding and compiling information. To minimize that influence, we attempted to appropriately sample participating and nonparticipating work groups and obtain information from a range of respondents. Case data were reviewed and summarized by more than one investigator, and results compared and discussed. The analytic generalizations concerning program revision may be relevant to others, but readers are cautioned to assess finding applicability for their settings. 

Worksite health promotion programs with proven efficacy seldom are widely implemented, and less than one percent of health promotion studies are categorized as diffusion research [21]. We believe that a component may be because marketing redesign and incorporation of contextual factors are seldom included in the efficacy to effectiveness pipeline. Including that step may assist others moving programs from knowledge to action and science to service. 

## 5. Conclusions

This study assessed an evidence-based program's translation among municipal fire departments. Many aspects identified as important in technology transfer were confirmed. Optimal implementation appeared to benefit from face to face recruitment, midlevel advocates, and capacity building among well-informed potential participants. A factor not often mentioned in health promotion dissemination is the need for planned beta testing as a translational step. Its importance was underscored by our findings, and its explicit inclusion would be a useful addition to models of adoption, implementation, and widespread use and enhance the ability of moving science-based programs to service. 

## Figures and Tables

**Table 1:** Translational framework.

	Features advancing adoption and implementation
New technology (product, program, or service)	Advantage over prior and existing programs [18]
	Ability to try and adapt the program [19]
	Feasibility in time and cost [18]
	Outcomes are observable locally and in other sites [18]
	Outcomes are reinforced [20]
	Proven efficacy and linkage with other satisfied users [18]
	Training and technical support available [21]

Users and organization	Local champion, change agents, and well-established person to person communication channels [18, 22]
	Compatibility with policies and values and climate where the innovation is expected, supported and rewarded [18, 22]
	Management support (usually mediated by resource availability) [22]
